# First report of *Anaplasma marginale* infection in goats, Brazil

**DOI:** 10.1371/journal.pone.0202140

**Published:** 2018-08-13

**Authors:** Nayara B. da Silva, Naomi S. Taus, Wendell C. Johnson, Anabela Mira, Leonhard Schnittger, Jessica D. M. Valente, Odilon Vidotto, Hayley E. Masterson, Thállitha S. W. J. Vieira, Massaro W. Ueti, Rafael F. C. Vieira

**Affiliations:** 1 Department of Veterinary Medicine, Universidade Federal do Paraná, Curitiba, Paraná, Brazil; 2 Animal Diseases Research Unit, Agricultural Research Service, U.S. Department of Agriculture, Pullman, Washington, United States of America; 3 Department of Veterinary Microbiology and Pathology, Washington State University, Pullman, Washington, United States of America; 4 Institute of Pathobiology, Center of Research in Veterinary and Agronomic Sciences, INTA-Castelar, Argentina; 5 Department of Preventive Veterinary Medicine, Universidade Estadual de Londrina, Londrina, Paraná, Brazil; Kansas State University, UNITED STATES

## Abstract

*Anaplasma marginale*, the causative agent of bovine anaplasmosis, is a tick-borne bacterium that causes significant economic losses for cattle industries and is increasingly being detected in other animal species. *Rhipicephalus microplus* is the main vector of this bacterium and may be found parasitizing small ruminants. In northeastern Brazil, multispecies grazing is a common family subsistence practice on smallholder farms possibly facilitating interspecies transmission of pathogens. Considering that *A*. *marginale* infection has been previously molecularly described in sheep, this study has aimed to estimate the prevalence of *A*. *marginale* and factors associated with the infection in goats from northeastern Brazil. A total of 403 goat blood samples were included in the study. An epidemiological questionnaire was applied to each farm owner addressing age, gender, presence of ticks and multispecies grazing. All samples were screened for *A*. *marginale-* and *A*. *ovis*-infection using primers targeting the *Anaplasma* spp. *msp4* gene. The identity of *A*. *marginale* in the blood was confirmed by PCR amplification of *msp5* followed by sequencing. *Anaplasma* spp. were differentiated by sequencing of the repeat region of the *msp1α* gene. For the statistical analysis the Chi-square or the Fisher’s exact test was used to verify association of the individual factors (age, gender, presence of ticks, and multispecies grazing) with *Anaplasma* spp. infection. We report the first molecular detection of *A*. *marginale* in goats from northeastern Brazil, based on *msp1α*, *msp4* and *msp5* gene sequencing analysis. Sequencing of the detected *A*. *marginale msp1α* gene revealed the F repeat. *Amblyomma parvum* and *R*. *microplus* were found feeding on animals.

## Introduction

*Anaplasma marginale*, the causative agent of bovine anaplasmosis, is a tick-borne bacterium that causes significant economic losses for cattle industries [1]. *A*. *marginale* infects cattle through tick transmission worldwide. The bacterium has been detected in non-bovine species, but the relevance of these other species in the ecology of bovine anaplasmosis is unknown. At least 20 ixodid tick species have been implicated in the transmission of *A*. *marginale*, including *Dermacentor* spp. and *Rhipicephalus* spp. [1].

*Anaplasma marginale* is found in regions where tick vectors are endemic. In tropical and subtropical regions, *Rhipicephalus microplus* is the vector of bovine anaplasmosis. Despite host specificity of *R*. *microplus* for cattle [2], this tick species may be found parasitizing small ruminants [3]. In Brazil, *R*. *microplus* is endemic [4] and hampers livestock production resulting in annual economic losses estimated at U$ 3.24 billion [5].

In northeastern Brazil, multispecies grazing is a common family subsistence practice on smallholder farms possibly facilitating interspecies transmission of pathogens. A study of co-grazing ruminants has shown a single *A*. *marginale* strain infecting coexisting cattle, buffalo and ticks [6]. *A*. *marginale* infection has been previously molecularly described in sheep from Iran [7]. However, to the best of our knowledge, *A*. *marginale* has never been detected in goats. This study aimed to estimate the prevalence of *A*. *marginale* and factors associated with the infection in goats from the State of Paraíba, northeastern Brazil.

## Material and methods

With the approval from the Ethics Committee for Animal Experimentation and Animal Welfare of the Universidade Federal da Paraíba (protocol 3305/14), a total of 403 blood samples from goats (368 females and 35 males), previously surveyed for other pathogens [8], were included in this study. All samples were collected in an anticoagulant tube containing ethylenediaminetetraacetic acid and stored at -80°C. An epidemiological questionnaire was given to each farm owner addressing age, gender, presence of ticks and multispecies grazing. The age of goats was stratified into groups of ≤ one year and > one year.

Genomic DNA was extracted from 200 μL of whole blood using a commercial kit (GE Healthcare, Little Chalfont, UK), according to the manufacturer’s instructions. Negative controls using ultra-pure water were performed in parallel to monitor cross-contamination in each batch of 30 samples.

PCR amplification of the caprine glyceraldehyde-3-phosphate dehydrogenase (GAPDH) housekeeping gene was done to verify successful DNA extraction, as previously described [9]. Samples were screened for *A*. *marginale-* and *A*. *ovis*-infection using previously described primers targeting the *Anaplasma* spp. *msp4* gene (≈870 bp) [10]. Amplified DNA fragments of the *msp4* gene from two *Anaplasma* spp. isolates were directly sequenced using the Sanger method, and analyzed sequences compared by BLASTn with those present in the GenBank^®^ database.

The identity of *A*. *marginale* in the blood was confirmed by PCR amplification of *msp5* followed by sequencing (GenBank accession numbers: MH037254-MH037263). *Anaplasma* spp. were differentiated by amplifying, cloning and sequencing of the repeat region of the *msp1α* gene as previously described [11]. The *msp1α* gene sequences obtained herein have been submitted to GenBank under the following accession numbers: MH590661-MH590670.

Either the Chi-square or the Fisher’s exact test was used to assess association of the individual factors such as age, gender, presence of ticks, and multispecies grazing including sheep and cattle with *Anaplasma* spp. infection. P-values were calculated and considered significantly different when p < 0.05. Data was compiled and analyzed by Epi Info™ Software (version 7.1.5, CDC).

## Results

**Fig 1:**
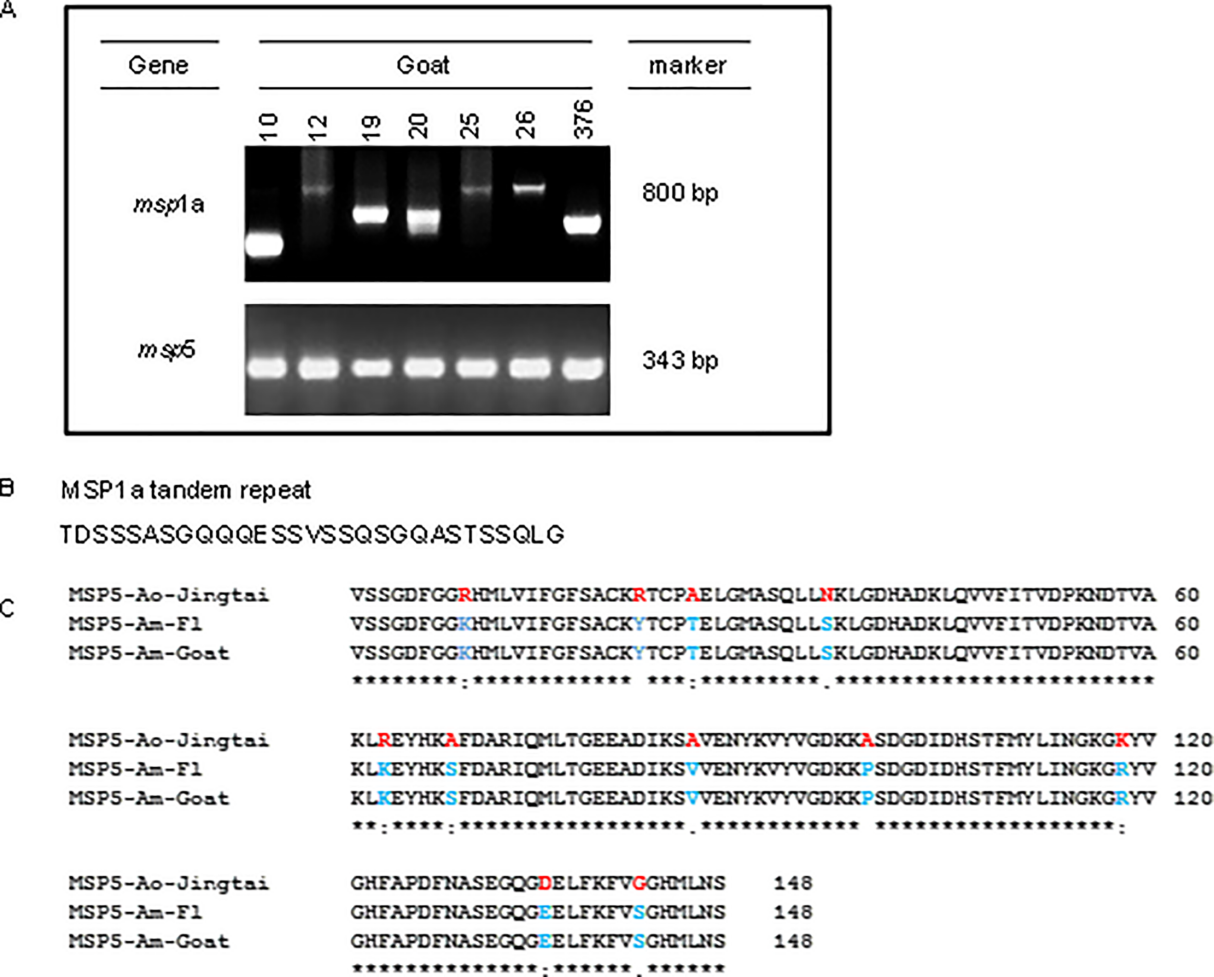
Detection of *A*. *marginale msp*1a and *msp*5 genes in infected goats. A) 2% agarose gel detecting *msp1a* and *msp5*, B) MSP1a tandem repeats and C) alignment of MSP5 between *Anaplasma ovis* (Ao Jintai), *Anaplasma marginale* Florida (Am-Fl) and *Anaplasma marginale* from a Brazilian goat (Am-Goat). Red indicates variation in the *A*. *ovis* MSP5 protein and blue highlights identical amino acids at the same location between Am-Fl and Am-Goat.

All samples have successfully amplified the GAPDH gene. Eleven out of 403 goats (2.73%; CI 95%: 1.53–4.82%) were positive for the *Anaplasma msp4* gene. All analyzed *Anaplasma* spp. *msp4* gene sequences showed ≥99% identity to multiple *A*. *marginale msp4* gene sequences deposited in GenBank (KX989533, AY283196, EU283844, AY702919, CP001079). Ten out of eleven samples were positive for the *Anaplasma msp*5 gene by nested PCR (Fig 1A), and sequencing of the *msp5* gene confirmed the presence of *A*. *marginale* sensu stricto (Fig 1C).

The rate of *A*. *marginale*-infection and corresponding estimated parameters for each of the evaluated potential risk factor is shown in Table 1.

**Table 1:** Prevalence of *Anaplasma marginale* in goats within each variable studied, Paraíba State, northeastern Brazil.

		*A*. *marginale–msp4* gene				
		+/n	(%)	OR	95% CI	p-value
**Age**	>1	10/337	2.97	1.9878	0.25–15.79	0.43814
	≤1*	1/66	1.52			
**Gender**	Female	10/368	2.72	0.9497	0.12–7.64	0.63673
	Male*	1/35	2.86			
**Presence of ticks**	Yes	3/26	11.54	6.0163	1.49–24.21	0.02788
	No*	8/377	2.12			
**Multispecies grazing**	Yes	11/304	3.62	†	†	0.04300
	No*	0/99	0.00			

Abbreviations: *msp*4, major surface protein 4; +, Number of positive animals; n, number of samples; OR, odds ratio; 95% CI, 95% confidence interval

†, not applicable

*, reference.

Tick-infested goats were six times more likely to be infected with *A*. *marginale* (p = 0.02788). The tick species feeding on the studied goats were identified as *Amblyomma parvum* (49/52, 94.23%) and *R*. *microplus* (3/52, 5.77%). All *A*. *marginale*-positive goats were found on farms with multispecies (sheep and cattle) grazing (p = 0.04300). To determine the *A*. *marginale* tandem repeats as previously described [12], we performed *msp1α* PCR (Fig 1A) and the amplicons were cloned and sequenced. The sequence results revealed MSP1a tandem repeat F in Brazilian goats (Fig 1B).

## Discussion

Although some goat *Anaplasma* MSP4 and MSP5 sequences previously reported in GenBank database have greater similarity to *A*. *marginale* than *A*. *ovis* (e.g. JN572928 and NZ_PKOE00000000), this study describes the first molecular report of *A*. *marginale* in goats based on *msp1a* gene sequence analysis. Anti-*Anaplasma* spp. antibodies have been identified in goats from northeastern Brazil [13]. However, direct molecular detection of *A*. *marginale* in small ruminants has been only reported in sheep from Iran [7]. *R*. *microplus* has been described as the main vector of *A*. *marginale* [1], yet no study has tested the vector competence of *A*. *parvum* for *A*. *marginale*. Previous studies have suggested that *Amblyomma* ticks may be involved in the transmission of *A*. *marginale* [6,14]. Further studies are necessary to evaluate if *A*. *parvum* is a competent vector of *A*. *marginale* in Brazil.

Co-grazing of goats, sheep and cattle is the most common practice of northeastern Brazil, since it allows a wider diversification of products for commercialization. A previous study in Rio de Janeiro State, southeastern Brazil, has shown that *A*. *marginale* strains identified in water buffaloes were closely related to *A*. *marginale* from cattle as determined by sequencing *msp1α* [6]. In the Brazilian goats, the detected *A*. *marginale* was also closely related to cattle species and all detected MSP1a displayed the F repeat. Due to the different number of tandemly repeated 29 amino acid units, the molecular weight of MSP1a varied between *A*. *marginale* isolates [15]. A previous report demonstrated individual animals had the same tandem repeat but different numbers of repeats ranging from 2 to 6 repeats [12]. In another study, molecular weight variation was used to differentiate and characterize geographic genotypes [15]. However, in both cases, sequences were used to determine *A*. *marginale* genotypes. In Brazil, six microsatellite genotypes have been reported, including repeats B, C, D, E, G and H, with genotype E being the most common in cattle [16,17]. Unfortunately, the nucleotide distance between the Shine-Dalgarno (SD) sequence and the translation initiation codon (ATG) could not be determined [18]. Thus, it is unknown whether other *A*. *marginale* genotypes infect goats. Additional studies are necessary to elucidate this question.

## Conclusions

This study provides the first insight into *A*. *marginale* infection in goats from Paraíba State, northeastern Brazil and demonstrates that goats may play a role in the epidemiology of *A*. *marginale* as a yet unrecognized reservoir. Competent ticks feeding on goats and cattle may transfer the pathogen between the two livestock species.
